# Trends in all cause and viral liver disease-related hospitalizations in people with hepatitis B or C: a population-based linkage study

**DOI:** 10.1186/1471-2458-11-52

**Published:** 2011-01-24

**Authors:** Heather F Gidding, Gregory J Dore, Janaki Amin, Matthew G Law

**Affiliations:** 1 National Centre in HIV Epidemiology and Clinical Research, The University of New South Wales, Sydney, Australia

## Abstract

**Background:**

Previous studies have reported an excess burden of cancer and mortality in populations with chronic hepatitis B (HBV) or C (HCV), but there are limited data comparing hospitalization rates. In this study, we compared hospitalization rates for all causes and viral liver disease in people notified with HBV or HCV in New South Wales (NSW), Australia.

**Methods:**

HBV and HCV notifications were linked to their hospital (July 2000-June 2006), HIV and death records. Standardized hospitalization ratios (SHRs) were calculated using rates for the NSW population. Random effects Poisson regression was used to examine temporal trends.

**Results:**

The SHR for all causes and non alcoholic liver disease was two-fold higher in the HCV cohort compared with the HBV cohort (SHRs 1.4 (95%CI: 1.4-1.4) v 0.6 (95%CI: 0.6-0.6) and 14.0 (95%CI: 12.7-15.4) v 5.4 (95%CI: 4.5-6.4), respectively), whilst the opposite was seen for primary liver cancer (SHRs 16.2 (95%CI: 13.8-19.1) v 29.1 (95%CI: 24.7-34.2)). HIV co-infection doubled the SHR except for primary liver cancer in the HCV/HIV cohort. In HBV and HCV mono-infected cohorts, all cause hospitalization rates declined and primary liver cancer rates increased, whilst rates for non alcoholic liver disease increased by 9% in the HCV cohort but decreased by 14% in the HBV cohort (*P *< 0.001).

**Conclusion:**

Hospital-related morbidity overall and for non alcoholic liver disease was considerably higher for HCV than HBV. Improved treatment of advanced HBV-related liver disease may explain why HBV liver-related morbidity declined. In contrast, HCV liver-related morbidity increased and improved treatments, especially for advanced liver disease, and higher levels of treatment uptake are required to reverse this trend.

## Background

Chronic infection with hepatitis B virus (HBV) or hepatitis C virus (HCV) is associated with increased morbidity and mortality. Several data linkage studies have reported an excess burden of hepatocellular carcinoma (HCC) and mortality, particularly from advanced liver disease [1-5]. They also highlight an increased disease burden associated with HBV/HIV, HCV/HIV and HBV/HCV co-infection [2-4,6]. These population-based studies outline the relative incidence of cancer and mortality, but there are limited data comparing the impact of HBV and HCV infection on hospitalization rates. The one published study compared the average number of hospitalizations for people notified with HBV and HCV with age and sex matched controls but did not examine rates for liver disease or trends over time [7].

There have been considerable advances in antiviral therapy for HBV and HCV since the mid-1990s. A population-based study examining trends in hospitalization rates, especially for liver-related admissions, may suggest an effect of improved therapy on disease burden. In particular, we hypothesize that trends in hospitalization rates for advanced HBV and HCV-related liver disease may be diverging, given that HBV therapy can be utilized in liver failure [8-10] and has been shown to reverse decompensated liver disease [11-14]. The aim of this study was, therefore, to compare the overall burden and trends in hospitalization rates for all causes and viral liver disease in people notified with HBV or HCV in New South Wales (NSW), Australia.

## Methods

### Data sources

The study cohorts comprised all people notified with acute or chronic HCV or HBV infection in NSW (population 7 million), as recorded on the NSW Notifiable Diseases Database (NDD) [15] between 1992 (when personal identifiers were first recorded) and 2006. These cohorts were further divided by their co-infection status by linkage of HCV and HBV datasets and obtaining HIV co-infection status by linkage to NSW data from the National HIV Registry (NHR) [16] and National AIDS Registry (NAR) [17]. The NDD, NHR and NAR records contain demographic information (including full name for NDD and name code [first two letters of surname and given name] for HIV and AIDS notifications) and variables for disease code and diagnosis date.

Hospital admissions for each cohort were obtained from the Admitted Patient Data Collection [15], a data base which covers all inpatient admissions from all public (including psychiatric) and private hospitals in NSW. The data are collected by financial year (1 July to 30 June) of separation (discharge, transfer, death, or change in admission type within the same hospital). Each admission includes demographic and administrative information and diagnosis and procedure fields coded at separation according to the 10th revision of the International Classification of Diseases-Australian Modification (ICD-10-AM). For our analysis, admissions were categorized by their principal (first) diagnostic code (used to record the main condition responsible for the stay in hospital). Three admission categories were examined: all causes, non alcoholic liver disease (ICD-10-AM codes K71.0-K77.8, diseases of the liver excluding alcoholic liver disease), and primary liver cancer (ICD-10-AM codes C22.0-C22.9, malignant neoplasms of the liver and intrahepatic bile ducts). Patient name has been recorded since 1 July 2000. For this reason, the study period was limited to separations from 1 July 2000 to 30 June 2006 (the most recent year data were available).

The Registry of Births Deaths and Marriages [15] is a registry of all deaths (based on receipt of a medical certificate of cause of death) in NSW and includes the date of death. We used date of death to censor a person's time at risk.

### Linkage process

Data linkage was carried out by the Centre for Health Record Linkage (CHeReL) [18]. The NDD, hospitalization and death data were linked using probabilistic record linkage methods and ChoiceMaker software [19]. A random sample of 1000 NDD records and their matched hospitalization and death data were reviewed by the CHeReL with a false positive rate of 0.2% and a false negative rate of less than 0.1%. Full name on the NDD data set was then recoded to name code before linkage with the AIDS/HIV registries using deterministic methods based on a 100% match on name code, date of birth and sex.

### Exclusions

For the study cohorts, we examined the distribution of hospitalizations around the time of diagnosis and determined that excluding admissions before or beginning within 14 days of the HCV or HBV diagnosis (or earliest of the two diagnosis dates if co-infected) was sufficient to reduce the bias towards higher rates of admission around the time of diagnosis, as previously noted [5] (n = 38 922, 15.3%). Consistent with this, cases were considered ineligible if they died before the start of the study period or within 14 days of their diagnosis date, or were diagnosed within 14 days of the end, or after, the study period (n = 6635, 4.8%). Cases missing their age or sex were excluded from the eligible cohort (n = 1406, 1.1%). Duplicate and nested hospital admissions (i.e. an admission within the date range of another admission for the same person) were removed from both the NSW population and cohort admitted patient data collections (0.7% of the 12 615 230 NSW admissions and 0.9% of the 262 834 cohorts' admissions) such that there was only one principal diagnostic code for each time period. Because of the extremely high frequency of admissions with a principal diagnostic code of extracorporeal dialysis (Z49.1; 9% of the NSW and 19% of the cohorts' admissions) we decided to exclude them from the analysis.

### Statistical analysis

Hospitalization rates were calculated using person-years at risk as the denominator. This was calculated for each person as the time 14 days after the HBV or HCV diagnosis or from the start of the study period (whichever was later) until the end of the study period or death (whichever came first). The time at risk included the time spent in hospital as the patient was still at risk of a new episode of care with a different principal diagnosis. Standardized hospitalization ratios (SHRs) were calculated by comparing numbers of admissions with those expected using hospitalization rates for the NSW population by 5 year age group, sex and calendar year. To account for the correlation between hospitalizations for the same person, 95% confidence intervals (CIs) for SHRs were calculated using the method by Stukel et al [20], and a random effects Poisson regression model [21] was used to estimate the mean change in hospitalization rates over time. An interaction term between disease cohort and time period was fitted to assess whether hospitalization rate changes differed by cohort, with the significance determined using the likelihood ratio test. Overall rates and SHRs for each admission category were presented for all of the disease cohorts, but trends by age group and over time were only presented where cohort sample sizes were sufficient.

### Ethics approval

Ethics approval for the use of these confidential health data was granted by the University of NSW and the NSW Population and Health Services Research Ethics Committee.

## Results

### Description of cohorts

There were 129 472 individuals notified with HBV, HCV or both infections eligible for the study. Of 86 501 HCV cases, 3.7% were also notified with HBV, 0.8% with HIV and 0.04% with both HBV and HIV. Of 46 188 HBV cases, 6.9% were also notified with HCV, 0.6% with HIV and 0.08% with both HCV and HIV. Compared with HBV mono-infected cases, HCV mono-infected cases were more likely to be male, have at least one hospitalization and die during the study period (Table 1). A similar trend was seen for cases co-infected with HBV and HCV, and especially HIV, compared with mono-infected cohorts.

**Table 1:** Characteristics of people notified with hepatitis B or C or co-infected with HIV in NSW, Australia

Attribute	HCV	HBV	HCV/HBV	HCV/HIV	HBV/HIV	HBV/HCV/HIV
Cohort size	82 601	42 694	3179	683	277	38
Age [years] at entry into study^† ^median (IQR)	37 (29-44)	35 (27-44)	35 (29-43)	36 (30-41)	37 (30-44)	35 (29-42)
Males N (%)	52 016 (63)	23 105 (54)	2314 (73)	622 (91)	263 (95)	35 (92)
Diagnosed with HCV/HBV during study period N (%)**^†^**	31 221 (38)	17 054 (40)	1479 (47)	272 (40)	93 (34)	24 (37)
Hospitalized during study period N (%)^**†**,‡^	33 152 (40)	12 381 (29)	1524 (48)	371 (54)	146 (53)	24 (63)
Died during study period N (%)**^†^**	2844 (3)	755 (2)	156 (5)	73 (11)	33 (12)	9 (24)
Total person years f/up^†^	404 471	206 943	15 023	3115	1278	152
Mean person years f/up per person^†^	4.9	4.8	4.7	4.6	4.6	4.0

^† ^Study period is the six years for which hospital data were available (1 July 2000-30 June 2006).

^‡ ^Excludes admissions for extracorporeal dialysis.

IQR, Interquartile range; f/up, follow-up.

### All cause hospitalizations

Compared with the NSW population, all cause hospitalization rates for the HCV mono-infection cohort were 42% higher than expected. In contrast, rates for the HBV mono-infection cohort were 37% lower than expected (Table 2). Thus, HCV mono-infection was associated with more than twice the burden of hospital-related morbidity compared with HBV mono-infection. Co-infection with either HIV or HBV/HCV added significantly to the burden but the additional burden was greater for HIV.

**Table 2:** Numbers and rates of hospitalization and standardized ratios for people with hepatitis B or C by HIV co-infection status, July 2000-June 2006

Type of admission	Disease	Admissions (n)	% All admissions	% All liver disease admissions^†^	Rate/1000	SHR	95% CI
All cause	HCV	131 707	100	-	325.6	1.4	1.4-1.4
	HBV	33 264	100	-	160.7	0.6	0.6-0.6
	HCV/HBV	6499	100	-	432.6	2.0	1.9-2.1
	HCV/HIV	2101	100	-	674.4	3.5	3.3-3.9
	HBV/HIV	616	100	-	482.2	2.4	2.0-2.7
	HCV/HBV/HIV	135	100	-	887.6	4.5	3.5-5.7
Non alcoholic liver disease	HCV	1632	1.2	27.3	4.0	14.0	12.7-15.4
	HBV	341	1.0	17.9	1.6	5.4	4.5-6.4
	HCV/HBV	111	1.7	30.8	7.4	24.6	15.7-38.5
	HCV/HIV	28	1.3	35.4	9.0	28.5	15.9-51.1
	HBV/HIV	18	2.9	45.0	14.1	40.0	19.4-82.6
	HCV/HBV/HIV	6	4.4	100.0	39.4	123.4	61.1-249.1
Primary liver cancer	HCV	625	0.5	10.4	1.5	16.2	13.8-19.1
	HBV	674	2.0	35.4	3.3	29.1	24.7-34.2
	HCV/HBV	50	0.8	13.9	3.3	31.9	17.5-58.3
	HCV/HIV	6	0.3	7.6	1.9	18.8	5.6-62.5
	HBV/HIV	12	1.9	30.0	9.4	71.1	18.3-276.7
	HCV/HBV/HIV	0	0.0	0.0	0.0	-	-

^† ^Percent of all liver disease-related admissions within each disease cohort: includes non alcoholic liver disease (ICD-10-AM codes K71-77), primary liver cancer (ICD-10-AM codes C22), viral hepatitis (ICD-10-AM codes B15-B19), and alcoholic liver disease (K70).

SHR, standardized hospitalization ratio; CI, confidence interval.

**Figure 1:**
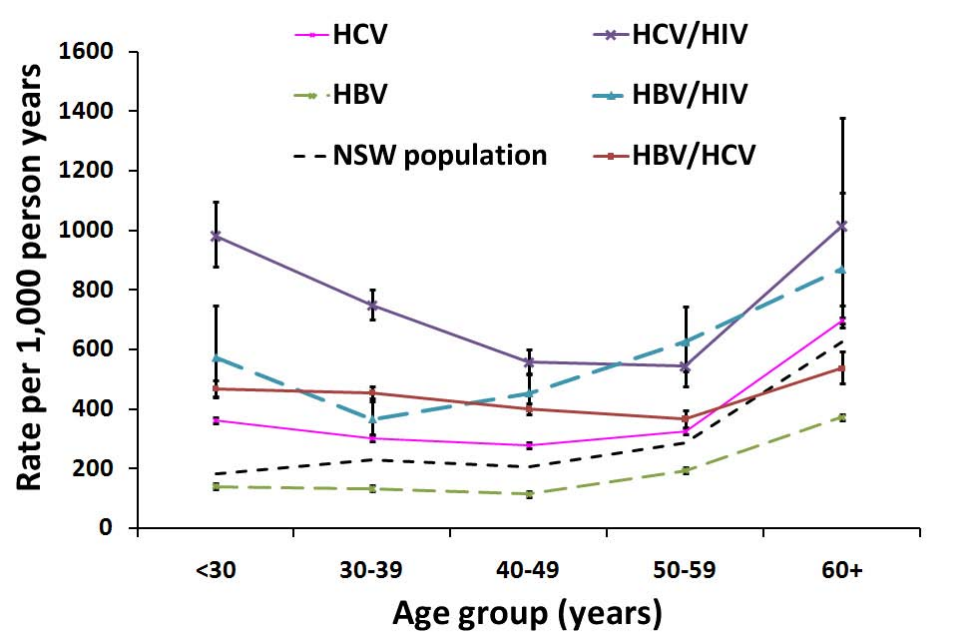
**All cause hospitalization rates for people notified with hepatitis B or C in NSW, Australia by their HIV co-infection status and age group**.

For all disease cohorts and the NSW population, all cause hospitalization rates were highest in the 60 years and over age group (Figure 1). However, unlike the NSW population (which had a secondary peak in 30-39 years) all disease cohorts had a secondary peak in less than 30 year olds, which was most pronounced for HIV co-infection cohorts. Therefore, excess rates (SHRs) for all disease cohorts were highest in the youngest age group examined and decreased with age, except for the HBV mono-infection cohort which showed no trend by age group.

Between 2000 and 2006, hospitalization rates for all causes declined significantly for HBV and HCV mono-infection cohorts (Table 3), with the annual rate of decline significantly greater for HBV than for HCV (*P *< 0.001). Hospitalization rates for the HCV/HIV and HBV/HCV cohorts showed no change over time, whilst rates for the HBV/HIV cohort declined by 15% between 2000-2003 and 2004-2006, although this decline was not statistically significant.

**Table 3:** Trends in hospitalization rates between 2000 and 2006 in people with hepatitis B or C by HIV co-infection status

Type of admission	Disease	Mean % change	95% CI	*P *value
All cause	HCV	-1.6^†^	-1.9 to -1.3	< 0.001
	HBV	-4.6^†^	-5.2 to -3.9	< 0.001
	HCV/HBV	-0.6^†^	-2.1 to 0.9	0.431
	HCV/HIV	0.3^‡^	-8.7 to 10.2	0.953
	HBV/HIV	-15.1^‡^	-29.4 to 2.0	0.081
Non alcoholic liver disease	HCV	9.4^†^	6.1 to 12.9	< 0.001
	HBV	-14.2^†^	-19.6 to -8.4	< 0.001
	HCV/HBV	7.9^†^	-4.3 to 21.6	0.213
	HCV/HIV	255.2^‡^	48.9 to 747.7	0.004
	HBV/HIV	-91.7^‡^	-98.9 to -35.7	0.017
Primary liver cancer	HCV	42.5^†^	33.8 to 51.8	< 0.001
	HBV	21.3^†^	14.4 to 28.6	< 0.001

^† ^Mean annual change in rate (adjusted for the correlation between hospitalizations for the same person).

^‡ ^Rate change between July 2000-December 2003 and January 2004-June 2006 (adjusted for the correlation between hospitalizations for the same person).

CI, confidence interval.

### Non alcoholic liver disease

The highest hospitalization rates and SHRs for non alcoholic liver disease were in the co-infected cohorts (Table 2). Compared with the HBV mono-infected cohort, the HCV mono-infected cohort had significantly higher hospitalization rates and SHRs, and non alcoholic liver disease accounted for a higher proportion of all hospitalizations and all liver disease-related hospitalizations in the cohort (Table 2; *P *= 0.002 and *P *< 0.001, respectively).

**Figure 2:**
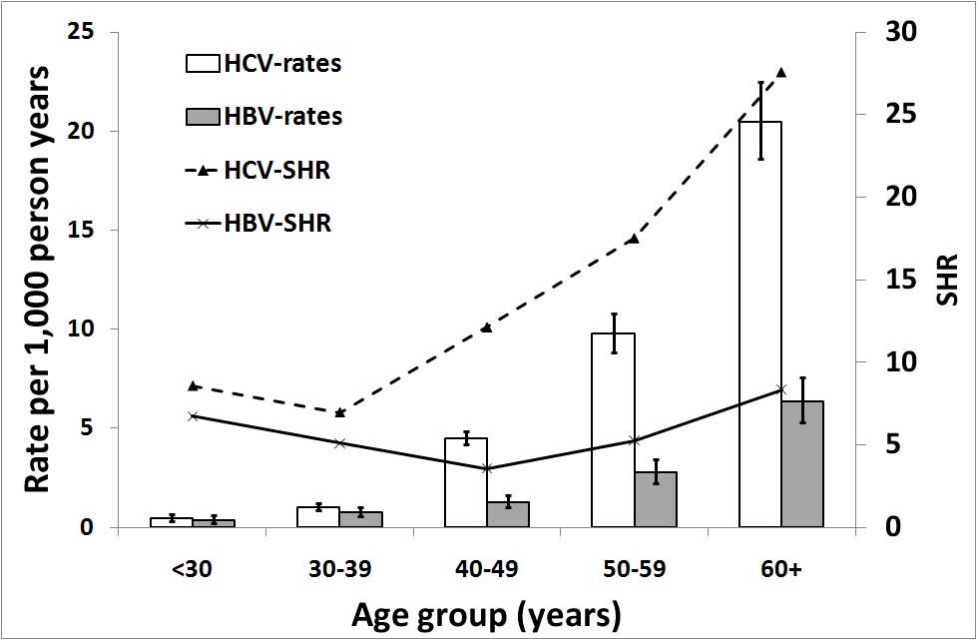
**Non alcoholic liver disease hospitalization rates for people notified with hepatitis B or C mono-infection in NSW, Australia by age group**. SHR, standardized hospitalization ratio.

For both the HBV and HCV mono-infection cohorts, hospitalization rates for non alcoholic liver disease increased with age and the highest rates and SHRs were in the 60+ age group (Figure 2). Rates and SHRs for HCV were higher than for HBV in all age groups, but more than three times higher for ages 40 years and over.

Hospitalization rates for non alcoholic liver disease demonstrated contrasting trends over time for the HBV and HCV mono-infected cohorts, with a significant increase in the HCV cohort and a significant decrease in the HBV cohort (Table 3). Similarly, within the HIV co-infection cohorts, the hospitalization rate increased for HIV/HCV but decreased for HIV/HBV.

### Primary liver cancer

The highest hospitalization rate and SHR for primary liver cancer was in the HIV/HBV co-infection cohort. However, due to the small sample size the SHR was not significantly different to that for other cohorts (Table 2). In the mono-infected cohorts, the SHR was significantly higher for HBV than HCV and primary liver cancer accounted for a higher proportion of all cause and all liver disease-related hospitalizations (*P *< 0.001 for both). This was in contrast to the comparison for non alcoholic liver disease.

**Figure 3:**
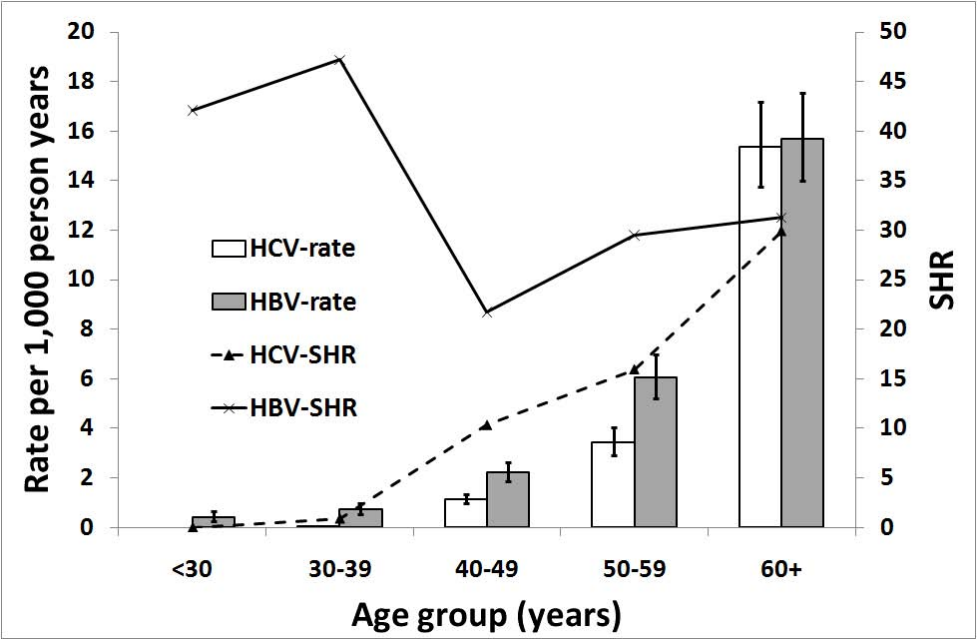
**Primary liver cancer-related hospitalization rates for people notified with hepatitis B or C mono-infection in NSW, Australia by age group**. SHR, standardized hospitalization ratio.

For both HBV and HCV mono-infection cohorts, hospitalization rates for primary liver cancer increased with age and the highest rates were in the 60+ age group (Figure 3). Rates and SHRs for HBV were higher than for HCV in all age groups except the 60+ age group.

Rates of hospitalization for primary liver cancer increased significantly between 2000 and 2006 for both the HBV and HCV mono-infection cohort (Table 3). However, rates increased significantly faster for the HCV than for the HBV mono-infection cohort (*P *= 0.028).

## Discussion

Our study revealed contrasting hospital-related morbidity among individuals diagnosed with HBV and HCV infection, and that hospitalization rates were consistently higher in all co-infected cohorts, particularly those with HIV. The all cause and non alcoholic liver disease hospitalization rates and SHRs were two-fold higher in the HCV mono-infection cohort compared with the HBV mono-infection cohort, with all cause rates for HBV mono-infection even lower than in the NSW population. In contrast, the HBV mono-infected cohort had a hospitalization rate and SHR for primary liver cancer close to two-fold higher than that for the HCV mono-infected cohort. Perhaps the most notable contrast, however, is that between 2000 and 2006 rates for non alcoholic liver disease increased significantly in the HCV mono-infected cohort but decreased significantly in the HBV mono-infected cohort. Trends that were further accentuated in the HIV co-infection cohorts and are suggestive of an impact of improved treatment for advanced HBV-related liver disease [8-10,12,14].

Contrasting all cause hospital-related morbidity between the HCV and HBV infected cohorts may relate to differing epidemiological patterns and health care utilization. In Australia, it is estimated that over 80% of HCV cases are in Australian-born injecting drug users, infected in their young adult years, with a smaller proportion among immigrants [22]. In contrast, it is estimated that over 50% of HBV cases are immigrants from HBV endemic countries in South-East and North-East Asia [23] with predominant perinatal or early childhood acquisition. A high proportion of the all cause hospitalizations amongst the HCV cohort in the less than 30 year age group has previously been reported to be lifestyle-related admissions such as drug and alcohol use [24]. Such factors may also explain the high rates of all cause hospitalization in young adults in the HIV co-infected cohorts. The lower rates of hospitalization in the HBV mono-infection cohort compared with the NSW population across all age groups may be due to a 'healthy immigrant' effect or lower levels of health care utilization amongst immigrant populations [25]. If it is the latter explanation, the comparative burden of morbidity associated with HBV may have been underestimated. Reasons for the downward trend in all cause hospitalization rates between 2000 and 2006 for both the HCV and HBV mono infected cohorts are unclear, as rates for the NSW population increased over the same period.

Rates of hospitalization for non alcoholic liver disease and primary liver cancer increased markedly with age in both the HBV and HCV infected cohorts, consistent with previous studies. In a systematic review, hepatic fibrosis progression was found to be non linear and significantly affected by duration of HCV infection [26]. In addition, a large cross sectional study showed that, regardless of the duration of HCV infection, the risk of cirrhosis increased significantly after the age of 50 years [27]. In HBV infection, risk of cirrhosis also increases with age, particularly after 50 years [28].

As expected, co-infection with HIV increased the burden of morbidity for both the HCV and HBV cohorts. Compared with the mono-infected cohorts, there was at least a two-fold increase in SHRs for all three admission types examined, except for primary liver cancer in the HCV/HIV cohort. HIV co-infection has been shown to be associated with an increased HBV and HCV viral load, a higher rate of fibrosis progression and increased risk of cirrhosis [29-35]. In addition, HIV co-infection has been associated with higher rates of HCC than HCV and HBV mono-infection [36].

The burden of non alcoholic liver disease was expected have increased during 2000-2006. This is because the disease cohorts are aging, as well as continuing to expand, albeit at a slower rate than in previous decades [37,38]. However, hospitalization rates for non alcoholic liver disease in the HBV and HBV/HIV cohorts declined during the review period, in contrast to the (expected) upward trend in the HCV and HCV/HIV cohorts. This is unlikely to be due to differential changes in case finding, which remained relatively constant over the review period, but may suggest an impact of HBV treatment at a population level.

There are several factors that may help to explain why HBV treatment has impacted on liver-related morbidity at a population level. First, therapy for HBV improved during the study period. Although lamivudine (available since 1998) can be successfully used to treat advanced HBV-related liver disease [12], the availability of adefovir (2004) and entacavir (2006), both of which have a higher genetic barrier to resistance than lamivudine, has generally lead to better maintenance of HBV DNA suppression, a further reduction in the risk of liver disease, and even reversal of decompensated liver disease [11,14]. Second, HBV treatment uptake improved over the study period [38]. Therefore, even though uptake overall remains relatively low in Australia for both HBV (5%) and HCV (1.7%) [38], patients presenting with HBV-related advanced liver disease would be expected to have high rates of treatment uptake and favourable outcomes [12,14]. This means that even low levels of HBV treatment uptake may reduce liver-related morbidity. In contrast, patients presenting with HCV-related advanced liver disease have either poor HCV treatment outcomes in the case of compensated cirrhosis [39], or are not eligible for treatment if decompensated cirrhosis is present.

The greater decline in non alcoholic liver disease hospitalizations for the HIV/HBV cohort compared with the HBV mono-infected cohort adds to the evidence for an impact of HBV therapy at a population level. Tenofovir was approved for HIV treatment in Australia in 2002, prior to approval of adefovir (2004), entecavir (2006) or tenofovir (2008) for HBV mono-infection. Tenofovir provides high and sustained levels of HBV DNA suppression in HIV/HBV and HBV populations, with no described resistance [10,40], and has been shown to lead to resolution of decompensated liver disease [13]. The earlier introduction and high uptake of tenofovir in HIV infected patients [38] is consistent with a more rapid decline in non alcoholic liver disease hospitalizations amongst the HBV/HIV co-infected cohort and with an impact of HBV therapy at a population level.

The contrasting trends in non alcoholic liver disease morbidity are consistent with trends for liver transplantation and mortality data. U.S. liver transplant waiting list data showed a 27% decline in HBV-related end stage liver disease registrations between 1999 and 2006 [41]. Similarly, Australian data for 1997-2006 show a decline in the number and proportion of transplant recipients with HBV-related cirrhosis, in contrast to the upward trend for HCV-related cirrhosis [37]. In addition, rates of non-HCC liver deaths in notified cases of HBV in NSW declined by 5% while there was no change in the HCV cohort [42]. The more pronounced trends for hospitalization rates seen in our study may be due to the relatively recent advances in antiviral treatment having a more immediate impact on morbidity than on mortality.

Rates of hospitalization for both HBV- and HCV-related primary liver cancer increased during 2000-2006, consistent with the increasing number of liver cancer diagnoses in Australia [43,44]. A further contribution to these trends may be increasing management options for primary liver cancer [45]. The higher hospitalization rates for primary liver cancer in the HBV-mono-infected cohort compared with the HCV mono-infected cohort may be because a higher proportion were infected at an early age (in their country of birth) and therefore have a greater cumulative risk of disease progression [46]. This may also explain the high SHRs in the less than 40 year age groups. However, only date of diagnosis (which may occur many years after the date of infection) is reported so we are unable to confirm what age cases were infected. Another reason for the difference may be that HBV-related liver cancer can occur in the absence of cirrhosis due to the direct oncogenic affects of the HBV virus [47]. Therefore, even if the overall cirrhosis risk is similar there may be a greater risk of liver cancer in HBV cases. Our results are consistent with other populations-based linkage studies in Australia which show higher mortality from, and incidence of, liver cancer in people with HBV compared to those with HCV [1,42].

The major strength of our study is that it is a large population-based linkage study including HIV co-infection status. By linkage of the cohorts to their hospital records we were also able to account for the correlation between hospitalizations for the same patient in our analyses of changes over time. The temporal trends presented here may therefore differ from the crude rate changes reported in other studies that were unable to account for within patient clustering or increases in the HCV and HBV infected population [48-50].

Population-based studies have some methodological limitations. In particular, caution needs to be exercised when comparing the different cohorts as some of the differences may be due to unmeasured confounding factors. Differences in the overall burden and trends over time may be due to variations in health care utilization rather than differences in actual morbidity. Second, while the aim of our study was to examine morbidity in cases of chronic HBV and HCV infection, more than 65% of HBV records and 77% of HCV records did not specify whether a case was acute or chronic and only 2% and 1% of cases, respectively, were recorded as newly acquired. For this reason all records were included, which would lead to an underestimate chronic HBV and HCV-related morbidity as some of the notified cases may have cleared their infection. However, it is estimated that 74% HCV infections are chronic [51] and most notified cases of HBV are also likely to be chronic given that a high proportion are thought to have acquired their infection in endemic countries during childhood [23]. Third, we were unable to exclude members of the disease cohorts from the reference (NSW) population. Although the cohorts only accounted for 1.5% of all cause hospitalizations in the NSW population, they accounted for one quarter of the liver cancer and 19% of the non alcoholic liver disease admissions. A comparison with the non infected population would therefore have yielded higher SHRs. Fourth, identifiers for matching HIV cases were not complete, so some of the HBV and HCV mono-infected cohort may include HIV positive cases, but again this would only lead to an underestimate of morbidity associated with HIV co-infection. Fifth, hospital data with identifiers for linkage were only available for a six year period from June 2000, so the trends identified here need to be monitored for a longer period of time. Sixth, there is likely to be some misclassification of alcoholic and non alcoholic liver disease diagnoses. However, this misclassification is likely to be non differential and there were no changes to the coding procedure over the period of the review. Therefore coding errors are unlikely to explain any divergent trends. Finally, for some of the analyses there were too few cases of HIV co-infected patients to examine trends.

## Conclusions

Our population-based study indicates that hospital-related morbidity overall and for non alcoholic liver disease is considerably higher in notified cases with HCV than in those with chronic HBV and that the gap in morbidity associated with these conditions is widening. The decline in HBV-related liver disease may, at least in part, be due to improved treatment of advanced HBV-related liver disease and the greater reduction in morbidity from non alcoholic liver disease in the HBV/HIV infected cohort supports this assertion. For the HCV infected cohort, liver-related morbidity is increasing and improved treatments, especially for advanced liver disease, and higher levels of treatment uptake are required to reverse this trend.

## Competing interests

The authors declare that they have no competing interests.

## Authors' contributions

The study was designed by HG and GD. Data extraction was undertaken by HG. The analysis was conducted by HG with assistance from ML and JA. The manuscript was drafted by HG with modifications by GD and ML. All authors read and approved the final manuscript.

## Pre-publication history

The pre-publication history for this paper can be accessed here:

http://www.biomedcentral.com/1471-2458/11/52/prepub
